# Prevalence of Malnutrition and Associated Factors among Under-Five Children in Pastoral Communities of Afar Regional State, Northeast Ethiopia: A Community-Based Cross-Sectional Study

**DOI:** 10.1155/2019/9187609

**Published:** 2019-06-02

**Authors:** Abel Gebre, P. Surender Reddy, Afework Mulugeta, Yayo Sedik, Molla Kahssay

**Affiliations:** ^1^Department of Public Health, College of Medicine and Health Sciences, Samara University, Semera, Afar Region, Ethiopia; ^2^School of Public Health, College of Health Sciences, Mekelle University, Mekelle, Tigray Region, Ethiopia; ^3^Afar Regional Health Bureau, Semera, Afar Region, Ethiopia

## Abstract

**Background:**

Malnutrition among children remains one of the most important causes of morbidity and mortality in the world. In Ethiopia, malnutrition is one of the most serious public health problem and the biggest in the world. This study aimed to assess the prevalence of malnutrition and associated factors among under-five children in pastoral communities of Afar Regional state, Northeast Ethiopia.

**Methods:**

A community-based cross-sectional study was conducted on 840 children aged 6–59 months from March 01–25, 2017. A multistage cluster sampling method was used to select the study participants. A structured questionnaire was used and anthropometric measurements were taken to collect data. EPI Data 3.1 and SPSS version 20.0 were used for data entry and analysis, respectively. Bivariate and multivariable logistic regression analysis was used to identify the factors associated with malnutrition. The statistical significance was declared at *p* value < 0.05 with 95% confidence intervals in the final model.

**Result:**

The study found the prevalence of wasting, stunting, and underweight was 16.2% (95% CI: 13.8–18.8%), 43.1% (95% CI: 39.8–46.5%), and 24.8% (95% CI: 21.9–27.8%), respectively. Family size (AOR = 2.72, 95% CI: 1.62–4.55), prelacteal feeding (AOR = 3.81, 95% CI: 1.79–5.42), and diarrhoea in the past two weeks (AOR = 4.57, 95% CI: 2.56–8.16) were associated with wasting. And sex of child (AOR = 1.98, 95% CI: 1.46–2.72), age of child ((12–23 months: AOR = 3.44, 95% CI: 2.24–5.29); (24–35 months: AOR = 3.58, 95% CI: 2.25–5.69); and (36–59 months: AOR = 4.42, 95% CI: 2.79–6.94)), and immunization status of child (AOR = 3.34, 95% CI: 1.31–4.81) were predictors for stunting. Moreover, mother's education (AOR = 4.06, 95% CI: 2.01–8.19), sex of child (AOR = 1.83, 95% CI: 1.29–2.94), prelacteal feeding (AOR = 2.81, 95% CI: 1.64–3.72), and immunization status of child (AOR = 3.17, 95% CI: 2.14–4.99) were significantly associated with underweight.

**Conclusions:**

This study indicated that child malnutrition was high among under-five children. Family size of five and above, receiving prelacteal feeding, and diarrhoea in the past two weeks were positively associated with wasting. Male child, increasing age of child, and not fully immunized child were positive predictors for increasing stunting. Maternal illiteracy, male child, prelacteal feeding, and not fully immunized child were factors affecting underweight. Promoting use of family planning, preventing diarrhoeal diseases, and vaccinating children integrated with the access of nutrition education programs are vital interventions to improve nutritional status of the children.

## 1. Background

Adequate nutrition is vital for healthy growth and development during childhood [1]. Malnutrition refers a pathological state resulting from relative or absolute deficiency or excess of one or more essential nutrients [2]. Wasting, stunting, and underweight are among those anthropometric indicators commonly used to measure under nutrition in a population of under-five children [3]. According to the World Health Organization (WHO), wasting, stunting, and underweight are defined as *Z*-scores less than −2 standard deviations of weight for height, height for age, and weight for age, respectively [4]. Wasting and stunting reflect acute and chronic exposures for nutritional deficiency, respectively. In addition, underweight reflects both acute and chronic exposures for nutritional deficiency [5, 6].

Malnutrition among children is one of the most important causes of morbidity and mortality in the world, particularly in developing countries [7]. It is the most important risk factor for the burden of disease causing about 300,000 deaths per year directly or indirectly responsible for more than half of the all deaths in children [8]. Globally, approximately 60 million and 13 million of children are affected with moderate and severe acute malnutrition, respectively [9]. Worldwide reports show that 21.9%, 13.4%, and 7.3% of under five years of age are stunted, underweight, and wasted, respectively [10]. The WHO also estimated that about 5.4 million under-five children die each year with 2.7 million deaths occurred in Sub-Saharan African countries including Ethiopia [11].

Many studies reported the health and physical consequences of child malnutrition include delaying their physical growth and motor development, lower intellectual quotient (IQ), greater behavioral problems, deficient social skills, and susceptibility to contracting diseases. Child malnutrition may also lead to higher levels of chronic illnesses in adult life which may have intergenerational effects, as malnourished females are more likely to give birth to low-weight babies [12, 13].

Malnutrition is not a simple problem with a single and simple solution. Multiple and hierarchically interrelated determinants are involved in causing malnutrition [14]. The most immediate determinants are inadequate dietary intake and disease which are themselves caused by a set of underlying factors: household food insecurity, poor maternal/child caring practices, and lack of access to basic health services including lack of safe water supply and unhealthy living environment such as open defecation [15, 16]. In turn, these underlying causes themselves are influenced by economic, political, and sociocultural conditions; national and global contexts; capacity, resources, environmental conditions, and governance [17].

The Ethiopian government has been implementing a number of strategies such as the 2004 National Strategy for IYCF practices, the 2005/2006 National Nutrition Strategy, and the 2008 National Nutrition Program [18–20]. Furthermore, the government has planned to reach the zero-level under nutrition by 2030 [21]. As a result, the country has demonstrated a promising progress in reducing child malnutrition over the past decades. Though the problem of under nutrition has decreased in the country, it still continues to be one of the countries with the highest burden of malnutrition among under-five children in the world causing a significant obstacle to achieving better child health outcomes [22]. The prevalence of underweight and stunting among young children are the highest among Sub-Saharan African countries. Moreover, 51% of under-five children's deaths are associated with malnutrition where its causes are multifaceted [21, 22].

According to the Ethiopian Demographic and Health Survey (EDHS) 2016 report, about 9.7% of the children were wasted, 28.7% of the children were underweight, and 44.4% of the children were stunted with wide regional variations [17, 22]. In Afar Regional State, the prevalence of wasting, stunting, and underweight among under-five children were 19.5%, 50.2%, and 40.2%, respectively, which is the highest as compared to the national average and across other regions [22]. Other earlier studies from other specific regions and localities of the country also indicated the prevalence of wasting in the range 11%–24%, stunting 35–49%, and underweight 21–48% [7, 23–27].

Although the prevalence of child malnutrition is relatively well documented among agrarian communities and urban dwellers of Ethiopia, evidence on the nature, prevalence, and factors affecting for child malnutrition in pastoralist communities is limited. And, national estimates are also usually not a reflection of the local estimate of child malnutrition. Afar regional state, where pastoralist communities mainly resides, has been identified as one of the hotspot regions in the country with high food insecurity, higher child malnutrition rates, and recurrent onset of droughts [28]. This indicted that investigating the problem and identifying its causative factors within this context is an important step to design appropriate strategies to mitigate the problem. Therefore, with this background in mind, this study aimed to assess the prevalence and associated factors with malnutrition among under-five children in Dubti district, Afar Regional State, Northeast Ethiopia.

## 2. Methods

### 2.1. Study Design and Setting

The study was conducted in Dubti district located at a distance of 850 km from the capital, Addis Ababa, and 80 km from the regional capital, Semera, in Northeast direction of Ethiopia. The district has 13 kebeles (the smallest administrative units). The total population of the district was estimated to be 65,314, of which 34,870 and 30,444 are males and females, respectively, as projected from the 2007 census of the district [29].

Most of the pastoral community's income depends on animal breeding. The district has two public health centers, ten health posts, and two private clinics providing health services. The most common childhood illnesses in the district are malnutrition, diarrhoeal diseases, malaria, pneumonia, and measles [28]. A community-based cross-sectional study was conducted from March 01–25, 2017 in the district to assess the prevalence and associated factors with malnutrition among under-five children in Dubti district, Afar Regional State, Northeast Ethiopia, through door-to-door visits. Each child had 6–59 months of age and his/her mother/care giver was chosen by systematic random sampling method and involved in the study.

### 2.2. Study Populations

All children aged 6–59 months old, and their mothers were the target for the study, whereas the study population consisted of a sample of all households with 6–59 months old children who were residing in the randomly selected kebeles. Those study participants who were residents of the study area for less than 6 months, children's mother who was seriously ill and difficulty to communicate, and children with physical deformities that hinder height measurements at the time of data collection were excluded from the study.

### 2.3. Sample Size and Sampling Procedure

Sample size was determined based on a single proportion population formula using *z*^2^ × *p* × *q*/*d*^2^ considering the following assumptions: 95% confidence level, estimated proportion (P) of wasting (19.5%), stunting (50.2%), and underweight (40.2%) taken from the previous EDHS report for Afar Regional State [22] and margin of error of 5%. Accordingly, the calculated sample size for prevalence of stunting was relatively largest (*n* = 384) and was taken as the sample size for this study. Considering design effect of 2% and 10% nonresponse rates, the final sample size was 844.

A multistage cluster sampling method was used to enroll the study participants from the pastoral communities. First, six out of the thirteen kebeles were selected by lottery method. Next, the total number of 6–59 months old children in the selected kebeles was taken from the respective households using the registration at health posts. Then, the calculated sample size (844) was proportionally allocated to the selected kebeles based on the total number of households with 6–59 months children in each kebele. Finally, participants were selected using systematic random sampling technique after identifying the first household randomly and proceeded to the second participant based on the K^th^ interval. Whenever there were two or more 6–59 months old children, the youngest child was selected to avoid recall bias.

### 2.4. Data Collection Procedures

The questionnaire was developed from the Ethiopian Demographic and Health Survey (EDHS) [22] and other relevant literatures based on the study objectives. The questionnaire was translated in to the local language, Afaraff, for data collection. The questionnaire consisted of socioeconomic and demographic factors, child feeding and caring practices, maternal health factors, environmental health related characteristics, and anthropometrics measurements.

A structured-interviewer-administered questionnaire in a face-to-face manner was used to collect the data from mothers of children 6–59 months of age. Six health-extension workers for data and two BSc nurse supervisors who are fluent speakers of the local languages (Afaraff) including the principal investigator were involved in the data collection process. Prior to the interview, verbal informed consent was obtained from all participants after explaining about the objective of the study, and it was confirmed that the information will be kept confidential.

### 2.5. Measurements

Anthropometric measurements such as weight and height of children were taken using the standard anthropometric measurement procedures outlined in the measurement guide prepared by the Food and Nutrition Technical Assistance (FANTA) project in 2007 [30]. Body weight was measured using a weighing scale in light clothing with no jackets or coats, shoes, and additional clothing to the nearest 0.1 kg on a new calibrated portable scale.

Height of children was measured using a portable stadiometer with no shoes; the shoulders, buttocks, and the heels touched the vertical stand with the head in Frankfurt's position to the nearest 0.1 cm. For children with 6–23 months of age, recumbent length and for children 24–59 months of age, standing height to the nearest 0.1 cm were measured. MUAC was measured by marking midway between shoulder tip and the elbow tip on the vertical axis of the upper arm with the arm bent at right angle and between the lateral and medial surface of the left arm [30, 31]. Age of each child was also collected from the mother and counter-checked using vaccination cards or other forms of informal recording. All anthropometric measurements were taken twice, and the average of the two measurements was calculated and recorded.

The 2006 WHO Anthro 3.2.1 software was used to convert weight, height, and age of child (months) into height-for-age (HAZ), weight-for-age (WAZ), and weight-for-height (WHZ) Z-scores to assess malnutrition taking sex in to consideration. Anthropometric classifications were based on global standards: <−3 SD, <−2 SD, and ≥−2 SD. Children with HAZ, WAZ, and WHZ below −2 SD of the median of reference population were considered as stunted, underweight and wasted, respectively. Children with HAZ, WAZ, and WHZ below −3 SD were also considered as severely stunted, wasted, and underweight, respectively.

Moreover, these variables were considered as the dependent variables during statistical analysis. The dichotomous variables stunting, underweight, and wasting were defined as 1 = for stunted and 0 = for not stunted, 1 = for underweight and 0 = for not underweight, and 1 = for wasted and 0 = for not wasted, respectively [16, 32].

Child feeding practices such as exclusive breastfeeding (EBF) were understood as feeding a child only breast milk without anything else for the first six months of life, with the exception of medicines for therapeutic purpose [33]. A 24-hour recall method (from sun rise to sun rise) was used to assess dietary diversity practices. This was based on the mother's recall of foods given to her child in the previous 24 hours prior to the interview date. Then, minimum dietary diversity was estimated using information collected from the 24-hour dietary recall.

Minimum dietary diversity was fulfilled if a child had received food from four or more food groups from the seven WHO food groups in the last 24 hours preceding the survey [34]. The seven food groups used included were grains, roots, and tubers; legumes and nuts; dairy products (milk, yogurt, and cheese); flesh foods (meat, fish, poultry, and liver/organ meats); eggs; vitamin-A rich fruits and vegetables; and other fruits and vegetables. Moreover, minimum meal frequency was fulfilled if breastfed child with the of age 6–8 months and 9–59 months received a minimum of two or three meals with one to two snacks and three or four meals with one to two snacks per day, respectively [33, 34].

Food security status of the households was determined based on nine standard Household Food Insecurity Access Scale (HFIAS) questions that were developed for this purpose by Food and Nutrition Technical Assistance (FANTA). The respondents were asked about the amount and variety of meal eaten and the occurrence of food shortage for the household members, causing them not to eat the whole day or eat at night only, in the past four weeks before the survey. Then, food-secure households were coded “1” and food-insecure ones “0” for further analysis [35, 36]. Child immunization status of children (full, partial, or never) was also checked by observing the immunization card, and if not available, mothers were asked to recall it. BCG vaccination was checked by observing scar on right (also left) arm.

### 2.6. Data Quality Control

To ensure data quality, the English version questionnaire was translated into the local language Afaraff and then back to English to maintain its consistency. Pretest was conducted on 42 subjects (5% of the sample) in kebeles not in the study for necessary modification. Two-day training was given to the data collectors and supervisors before the actual date of data collection. Continuous supervision was done by the supervisors and principal investigator on daily bases.

### 2.7. Statistical Analysis

Data entry and analysis was done using EPI data 3.1 and SPSS version 20.0, respectively. Anthropometric indices were calculated using the 2006 WHO Anthro 3.2.1 Software. Descriptive analysis was used to describe the percentages and frequency of sociodemographic characteristics and other relevant variables in the study. Bivariate and multivariable logistic regression analysis was used to identify the factors associated with child malnutrition. Both crude and adjusted odds ratios together with their corresponding 95% confidence intervals were computed to see the strength of association between the outcome and independent variables.

All independent variables that were associated with the outcome variables (wasting, stunting, and underweight) in bivariate analysis (with *p* value < 0.20) were included in the final multivariable logistic analysis. A *p* value of < 0.05 was considered to declare the result as statistically significant. The Hosmer–Lemeshow test was performed for model fitness in the final model, and *p* value > 0.05 was considered a good fit. The result was presented in text, tables, and graphs based on the types of data.

### 2.8. Ethical Considerations

Ethical clearance was obtained from the College of Health Sciences of Samara University, Research and Ethical Review Committee (RERC). Then, officials at different levels in the study area were communicated through letters from Afar National Regional Health Bureau. Letters of permission were obtained from district administrative and health offices. Verbal informed consent was obtained from each participant prior to the interview after explaining the purpose of the study.

## 3. Results

### 3.1. Demographic and Socioeconomic Characteristics

In this study, the final analysis included 840 mothers with their children aged 6–59 months, making the response rate of 99.5%. Of the total respondents, 806 (96.0%) and 804 (95.7%) of them were Afar in their ethnicity and Muslims in their religion, respectively. Majority of respondents, 758 (90.2%), were currently married. 726 (86.4%) of respondents were illiterate, and 678 (79.8%) of them were housewives by occupation (Table 1).

Majority, 624 (74.3%) of households were headed by fathers, whereas 421 (67.5%), 123 (19.7%), and 49 (7.8%) of them were pastoralists, agropastoralists, and employed, respectively. Of the total interviewed households, about 724 (86.2%) of them owned livestock. 591 (70.5%) the households had more than five family size, whereas half of the households, 424 (50.5%), had at least two under-five children.

### 3.2. Characteristics and Caring Practices of Children

Among the children participated in this study, 476 (56.7%) and 364 (43.3%) were males and females, respectively, where 486 (57.8%) fell in the age group of 6–23 months. The mean age ± SD of children were 17.6 ± 5.3 months. Of the studied children, 352 (41.9%) of them started breastfeeding immediately after birth within one hour. About 352 (41.9%) of children received prelacteal feeding; animal milk was the most predominant prelacteal food given, which accounts for 262 children (74.4%) followed by butter, 69 (19.6%) (Table 2).

In addition, 245 (29.9%) of studied children were exclusively breastfed for six months. About, 105 (12.5%) children started complementary feeding at 6 months and 328 (39.0%) of children feed more than three times per day. Concerning vaccination, 588 (70.0%) of the children did not receive full vaccine, 108 (12.9%) and 100 (11.9%) of children had diarrhoea and fever, respectively, in the past two weeks prior to the study period.

### 3.3. Maternal and Environmental Health Characteristics

Among the total mothers interviewed, 494 (58.8%) of them attended antenatal care visit child and 357 (42.5%) of mothers delivered at health institution for their index child. The source of drinking water for 410 (48.8%) of households was protected source (Table 3).

With regard to the presence of latrine, 362 (43.1%) of households had latrine, whilst 766 (91.2%) of households used open field to dispose solid waste. Moreover, 720 (85.7%) of respondents were washed their hand before preparing food, followed by 280 (33.3%) after latrine use.

### 3.4. Prevalence of Malnutrition among Children

The analysis of the three anthropometric indices revealed that the prevalence of wasting, stunting, and underweight were 16.2% (95% CI: 12.9%–19.9%), 24.8% (95% CI: 20.8%–29.1%), and 43.1% (95% CI: 38.4%–47.9%), respectively. Moreover, the prevalence of severe wasting, stunting, and underweight among the children was 5.7%, 16.7%, and 13.1%, respectively (Table 4).

**Figure 1:**
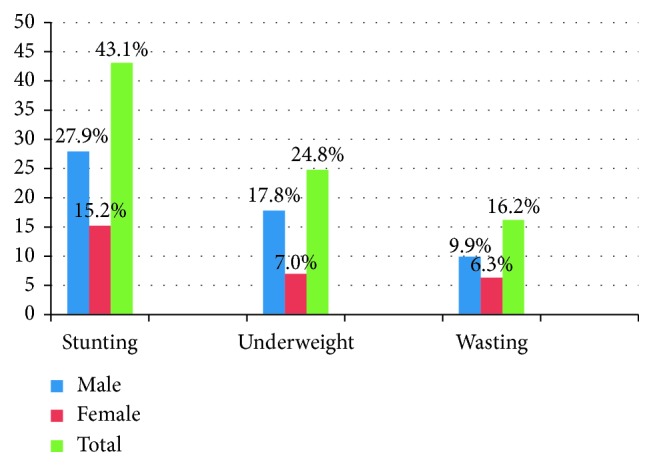
Prevalence of malnutrition by sex among under-five children in Dubti district, Afar Regional State, Northeast Ethiopia, 2017 (*n* = 840).

The prevalence of stunting was recorded in 234 (27.9%) male children, which was higher compared to females, 128 (15.2%). Similarly, underweight was higher among males, 149 (17.8%), than females, 59 (7.0%). However, wasting was slightly higher in males, 83 (9.9%) than in females, 53 (6.3%) (Figure 1).

**Figure 2:**
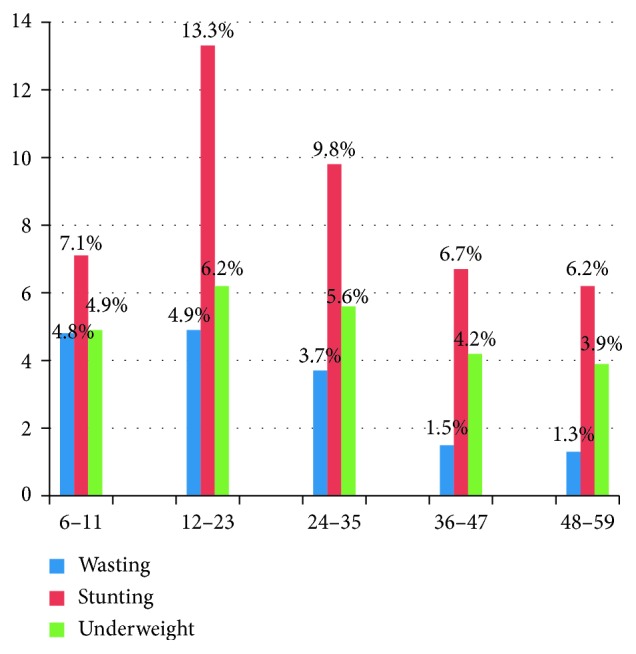
Prevalence of malnutrition by age group (months) among under-five children in Dubti district, Afar Regional State, Northeast Ethiopia, 2017 (*n* = 840).

Comparing age groups, the highest prevalence of wasting was seen among children aged 12–23 months with prevalence of 4.9% and the lowest among children aged 48–59 months with prevalence of 1.3%. Similarly, the highest prevalence of stunting, 13.3%, was seen among children aged 12–23 months and the lowest prevalence, 6.2%, in children aged 48–59 months. Moreover, the prevalence of underweight was lower for younger children and increases around 6–11 months of age making the highest in children aged between aged 12–23 months (Figure 2).

### 3.5. Factors Associated with Malnutrition of Children

#### 3.5.1. Factors Associated with Wasting

Among the variables entered into bivariate logistic regression analysis, mother's occupation, family size, place of delivery, initiation of breastfeeding, prelacteal feeding, diarrhoea in the last two weeks, and presence of latrine were associated with wasting at *p* value < 0.25. After controlling for potential confounders, the final multivariable logistic regression analysis revealed that family size (AOR = 2.72, 95% CI: 1.62–4.55), prelacteal feeding (AOR = 3.81, 95% CI: 1.79–5.42), and presence of diarrhoea in the past two weeks prior to the study (AOR = 4.57, 95% CI: 2.56–8.16) were the independent predictors for child wasting (Table 5).

Therefore, children living in households with greater than or equal to five family members were 2.72 times more likely to be wasted than those children living in households with less family members (AOR = 2.72, 95% CI: 1.62–4.55). Children who received prelacteal feeding at the time of birth were 3.8 times more likely to be wasted than those children who did not receive prelacteal feeding at the time of birth (AOR = 3.81, 95% CI: 1.79–5.42). Children who had diarrhoea in the past two weeks prior to the study were 4.6 times more likely to be wasted than those children without diarrhoeal disease (AOR = 4.57, 95% CI: 2.56–8.16).

#### 3.5.2. Factors Associated with Stunting

In the bivariate logistic regression model, sex and age of child, prelacteal feeding, child immunization status, timely complementary feeding, diarrhoea in the last two weeks, and presence of latrine were associated with stunting. However, sex of index child (AOR = 1.98, 95% CI: 1.46–2.72), age of index child ((AOR = 3.44, 95% CI: 2.24–5.29), (AOR = 3.58, 95% CI: 2.25–5.69), and (AOR = 4.42, 95% CI: 2.79–6.94)), and child immunization (AOR = 3.34, 95% CI: 1.31–4.81) were independent predictors for child stunting in the final multivariable logistic regression analysis (Table 6).

Therefore, male children had 1.9 times higher risk to develop stunting as compared to female children (AOR = 1.98, 95% CI: 1.46–2.72). It was also observed that children in the age ranges of 12–23, 25–34, and 35–59 months old were 3.4, 3.6, and 4.4 times more likely to be stunted than those of children in 6–11 months of age ranges, respectively, ((AOR = 3.44, 95% CI: 2.24–5.29), (AOR = 3.58, 95% CI: 2.25–5.69), and (AOR = 4.42, 95% CI: 2.79–6.94)). And, not fully immunized children were 3.3 times more likely to be stunted than fully immunized children (AOR = 3.34, 95% CI: 1.31–4.81).

#### 3.5.3. Factors Associated with Underweight

The bivariate logistic regression analysis showed that mother's education, mother's occupation, family monthly income, sex of index child, initiation of breastfeeding; prelacteal feeding and child immunization status were associated with underweight. However, after controlling for potential confounders, the final multivariable logistic regression model analysis showed that mother's education (AOR = 4.06, 95% CI: 2.01–8.19), sex of index child (AOR = 1.83, 95% CI: 1.29–2.94), prelacteal feeding (AOR = 2.81, 95% CI: 1.64–3.72), and child immunization (AOR = 3.17, 95% CI: 2.14–4.99) were independent predictors for underweight (Table 7).

Therefore, the risk of developing underweight among children who had illiterate mothers was 4.1 times higher than that of children whose mothers were literate (AOR = 4.06, 95% CI: 2.01–8.19). Male children were 1.8 times more likely to be underweight than female children (AOR = 1.83, 95% CI: 1.29–2.94). It was also observed that the likelihood of being underweight among children who received prelacteal feeding at the time of birth was 2.8 times higher than that of children who did not receive prelacteal feeding at the time of birth (AOR = 2.81, 95% CI: 1.64–3.72). And, not fully immunized children had about 3.2 times higher risk to develop underweight as compared to fully immunized children (AOR = 3.17, 95% CI: 2.14–4.99).

## 4. Discussions

Child malnutrition continues to be a major public health problem in developing countries including Ethiopia. Children are most vulnerable to malnutrition because of low dietary intakes, infectious diseases, lack of appropriate care, and inequitable distribution of food within the household in developing countries [37, 38]. Therefore, the current study aimed to assess the prevalence and associated factors with malnutrition (wasting, stunting, and underweight) among under-five children in Dubti district, one of the pastoralist communities of Afar Regional State, Northeast Ethiopia.

The current study revealed that 16.2% (95% CI: 13.8–18.8%), 43.1%(95% CI: 39.8–46.5%) and 24.8% (95% CI: 21.9–27.8%) of the under-five children were wasted, stunted and underweight, respectively in the study area, which were found to be very high according to WHO classification [33]. Regarding the associated factors of malnutrition, analysis of this study indicated family size, prelacteal feeding practice and diarrhoeal morbidity in the past two weeks were significantly associated with wasting. Furthermore, sex of child, age of child and child immunization status were the independent predictors for stunting. According to this finding, mother's education, sex of child, prelacteal feeding, and child immunization status were significantly associated with underweight in the study community.

In this study, the prevalence of wasting (16.2%) was similar with the regional figure, 19.5% [22] and Hidabu Abote district, Ethiopia, 16.7% [12]. But, it was much higher than the national figure, 9.7% [22], and various studies done in Ethiopia like Damot district, 9% [14], Metekele zone, 10.2% [39], Haramaya district, 10.7% [24], and other countries including Kenya, 2.6% [40], and Nigeria, 8.8% [41], which was an alarming case to increase risk of deaths among children. Furthermore, it also was lower than the study done in the pastoral communities of Dollo Ado district, Eastern Ethiopia, 42.3% [26]. Wasting is an indicator of acute malnutrition that can occur due to recent infection or weight loss due to periodical variation of food supply. The possible explanation for this difference might be due to a difference in the socioeconomic, recurrent drought, agroecology, and feeding habits of the study population. An additional explanation could also be that the study was conducted during drought season, when milk production, the most food in the community was scarce.

In this study, the prevalence of stunting (43.1%) was similar to the national figure, 44.4% [22]. But, it was much higher than other studies done in Ethiopia like Somali region, 22.9% [31], Dollo Ado district, 34.4% [26], Gumbrit district, 24.0% [42], and other countries including Kenya, 28.9% [43], Sudan, 24.9% [44], and Mongolia [45]. It was also lower than the regional figure, 50.2% [22], Libo-kemekem, Ethiopia, 49.4% [46], and India, 74.2% [47]. Stunting showed a failure to get adequate food over long period and affected through infections. Despite little improvements from 2016 EDHS report, the current prevalence of stunting is still a public health problem of the area. The possible explanation for this difference in prevalence could be due to a difference in the socioeconomic, agricultural productivity, food insecurity at household level, and nomadic nature of the population. An additional explanation for this could also be due to a difference in cultural and child feeding habits, study setting, and periods of the study.

In this study, the prevalence of underweight (24.8%) was found to be lower than the national figure, 28.7%, and regional figure, 40.2%, of EDHS report [22] and other studies done in Ethiopia such as West Gojam zone, 49.2% [23], Dollo Ado district, 47.7% [26], Sudan, 52.0% [48], India, 32.4% [49], and Pakistan, 39.5% [50]. However, it was also relatively similar with the findings from Haramaya, district, Ethiopia, 21.0% [24], Bahir Dar, Ethiopia, 22.1% [51], and Nigeria, 24.6% [41], and higher than the results in Kenya, 11.8% [40]. The possible explanation for the improvement in weight for age could be due to current efforts of the health sector to improve health intervention through health extension workers at the community level. An additional explanation could also be due to the fact that the Ethiopian health extension program has included underweight as indicator for child growth monitoring and promotion at the community level.

In the current study, mother's educational status was a strong predictor for high prevalence of underweight. And, the risk of developing underweight among children who had illiterate mothers was 4.1 times higher than children whose mothers were literate which showed that as mother's educational level increased, child nutritional well-being also increases. This might be due to the fact that educated mothers would have proper management of resources, practice better health promoting behaviors, and might develop better children centered caring practices. This result is in line with other studies conducted in Hawassa Zuria, Ethiopia [25], Dollo Ado district, Ethiopia [26], Rwanda [52], and Iran [53].

In the current study, sex of index child was strongly associated with both stunting and underweight. The likelihood of being both stunted and underweight among male children were 1.9 and 1.8 times higher when compared to female children. This could partially be explained due to the fact that boys are more vulnerable to health inequalities than their female counterparts in the same age groups [26]. Many studies in Ethiopia, Dollo Ado district [26], Bule Hora district [54], and Benna Tsemay district [55], and other developing countries, Kenya [40], Indonesia [56], and Pakistan [50], reported similar results. A systematic review conducted in Ethiopia and other developing countries also showed that male children were highly vulnerable to malnutrition when compared with female children due a difference in the frequency of eating, energy expenditure, and exposure to health problems than female children [57, 58].

In the current study, household family size was associated with wasting among study children. Children living in households with greater than or equal to five family members had 2.7 times higher risk to be wasted than children living in households with less than five family members. This might be due to fact that families with more children might experience more economic strain for food consumption; hence, they might be more likely to suffer from poor nutritional status since the available food is shared by all members. This is in agreement with other studies done in Ethiopia, Libo-Kemekem district [46] and Benna Tsemay district [55], and other countries like Bangladesh [9].

In the current study, age of the child was another factor associated with stunting. Children in the age range of 12–23, 25–34, and 35–59 months were about 3.4, 3.6, and 4.4 times, respectively, higher risk to be stunted than children of 6–11 months of age ranges. This might due to the fact that stunting is a cumulative process that can begin in utero and continues to about five years after birth [23]. The other possible reason also might be caring practices usually tend to decrease when children grown-up than infants and shifted to adult foods. Similar results have been reported from Libo-kemekem district, Ethiopia [46], Hawassa Zuria, Ethiopia [25], Kenya [40], and India [59]. However, an opposite result was still reported from studies done in Shey Bench district, Southwest Ethiopia [37], West Gojam zone, Ethiopia [23], and Vietnam [60] indicated that highest risk of stunting occurs in the youngest age group.

In the current study, prelacteal feeding was another factor strongly associated with both wasting and underweight. This study showed that wasting and underweight among children who received prelacteal feeding at the time of birth were 3.8 and 3.2 times, respectively, higher than children who did not receive prelacteal feeding at the time of birth. This might due to its negative impact on breastfeeding, and when children are not breastfed appropriately, they are at higher risk of developing undernourishment [23]. Studies conducted in Bule Hora district, Ethiopia [54], Mai-Aini Eritrean Refugees' Camp, Ethiopia [61], and Hidabu Abote district, Ethiopia [12] have revealed consistent with current study.

In the current study, immunization status of children had a significant association with increased risk of high prevalence of stunting and underweight. Nonimmunized children were 3.3 and 3.8 times, respectively, more likely to be stunted and underweight than immunized children. This could partially be explained that nonimmunized children could be at risk of many vaccine preventable diseases such as diarrhoea and respiratory infections, which might result in depleting nutrients from the body. This finding was in line with other studies conducted in Ethiopia, Metekele Zone [39], Shinille district [62], EDHS report [22], and other countries such as Zambia [63].

In the current study, presence of diarrhoeal disease was significantly associated with increasing prevalence of wasting. Children who had diarrhoeal disease in the past two weeks prior to the study were 4.6 times more likely to be wasted than those children without diarrhoeal disease. This might be due to the fact that diarrhoea may result in lower appetite, poor digestion, and malabsorption which leads to malnutrition. The other possible reason also might be that malnourished children would have more diarrhoeal episodes and a child with diarrhoea losses weight and can quickly become malnourished. This result was in line with other findings conducted in Damot district, Ethiopia [16], and Vadodara, India [49].

### 4.1. Limitation of the Study

As the study involved a single cross-sectional design, causal inference might not be strong between the dependent and independent variables. There might also be the possibility of recall and reporting bias in some infant and young child feeding (IYCF) indicators such as breastfeeding patterns, dietary diversity, and child's history of illness events happening in the past.

## 5. Conclusions and Recommendations

The present study revealed that the prevalence of wasting, stunting, and underweight were about 16.2% (95% CI: 13.8–18.8%), 43.1% (95% CI: 39.8–46.5%), and 24.8% (95% CI: 21.9–27.8%), respectively, in the study area. This result indicated the prevalence of child malnutrition (wasting, stunting, and underweight) was a serious public health problem in the pastoral community according to the WHO classification for public health significance. According to analysis of independent variables with the outcome variables, households having a family size of five or more, receiving prelacteal feeding, and presence of diarrhoeal disease in the past two weeks were the independent predictors for increasing wasting. Being male child, increasing age child, and not fully immunized child were the independent predictors for increasing stunting.

Moreover, the independent predictors positively *y* associated with increasing underweight were being illiterate mother, being male child, prelacteal feeding practices, and not fully immunized child. Promoting use of family planning, preventing diarrhoeal diseases, and vaccinating children integrated with the access of nutrition education programs are vital interventions to improve nutritional status of the children. A due emphasis should also be given to strengthen the health extension program to improve and provide participatory nutrition education to create awareness and to develop behavior change communication for better child feeding and caring practices in the pastoral community.

## Figures and Tables

**Table 1:** Demographic and socioeconomic characteristics of respondents in Dubti district, Afar Regional State, Northeast Ethiopia, May, 2017 (*n* = 840).

Characteristics	Categories	Frequency (*n*)	Percent (%)
Mother's age (in years)	15–19	76	9.0
	20–24	226	26.9
	25–29	340	40.5
	30–34	110	13.1
	35–39	47	5.6
	≥40	41	4.9

Mother's marital status	Currently married	758	90.2
	Divorced	30	3.5
	Widowed	35	4.1
	Others	19	2.2

Mother's ethnicity	Afar	806	96.0
	Others^*∗∗*^	34	4.0

Mother's religion	Muslim	804	95.7
	Others^*∗∗*^	36	4.2

Mother's education	Illiterate	726	86.3
	Primary (grade 1–8)	71	8.5
	Secondary (grade 9–12)	29	3.5
	Above secondary (grade 12^+^)	14	1.7

Mother's occupation	Housewife	678	80.7
	Herding livestock	92	10.9
	Government employee	38	4.5
	Others	32	3.9

Head of household	Father	624	74.3
	Mother	216	25.7

Total family size	<5	249	29.6
	≥5	591	70.4

Number of under-five children	1	326	38.8
	2	424	50.5
	≥3	90	10.7

Family monthly income (ETB)	<500	665	79.2
	500–1000	103	12.3
	>1000	72	8.5

Household food security	Secured	267	31.8
	Not secured	573	68.2

Ownership of livestock	Yes	724	86.2
	No	116	13.8

Others include ^*∗*^Amhara, Tigray, Oromo; ^*∗∗*^Orthodox, Protestant. ETB = Ethiopian birr.

**Table 2:** Characteristics and caring practices of under-five children in Dubti district, Afar Regional State, Northeast Ethiopia, May 2017 (*n* = 840).

Characteristics	Categories	Frequency (*n*)	Percent (%)
Child's sex	Male	476	56.7
	Female	364	43.3

Child age in months	6–11	254	30.2
	12–23	232	27.6
	24–35	154	18.3
	36–47	104	12.4
	48–59	96	11.4

Birth order	1	94	11.2
	2–3	422	50.2
	4–5	194	23.1
	≥6	130	15.5

Initiation of breastfeeding	Within one hour	352	41.9
	Hours later	290	34.5
	Days later	198	23.6

Received prelacteal feeding	Yes	352	41.9
	No	488	58.1

Type of prelacteal food (*n* = 352)	Animal milk	262	74.4
	Butter	69	19.6
	Water	15	4.3
	Others	6	1.7

Avoiding colostrum	Yes	384	45.7
	No	456	54.3

Exclusive breastfeeding	Yes	245	29.9
	No	595	70.1

Currently breastfeeding	Yes	286	34.0
	No	554	66.0

Timely complementary feeding started	Yes	105	12.5
	No	735	87.5

Materials used to feed	Spoon	388	46.2
	Cup	172	20.5
	Hand	242	28.8
	Bottle	38	4.5

Dietary diversity score	<4	138	16.4
	≥4	296	35.2

Child immunization	Yes	252	30.0
	No	588	70.0

Diarrhoea preceding two weeks	Yes	108	12.9
	No	732	87.2

Fever preceding two weeks	Yes	100	11.9
	No	740	88.1

**Table 3:** Maternal and environmental health characteristics of respondents in Dubti district, Afar Regional State, Northeast Ethiopia, May 2017 (*n* = 840).

Characteristics	Categories	Frequency (*n*)	Percent (%)
Antenatal care follow-up (index child)	Yes	494	58.8
	No	346	41.2

Place of delivery for (index child)	Home	483	57.5
	Health institution	357	42.5

Hand washing practice of mother^*∗*^	After latrine use	280	33.3
	Before preparing food	720	85.7
	Before serving food	224	26.7

Source of drinking water	Unprotected source	410	48.8
	Protected source	430	51.2

Presence of latrine	Yes	362	43.1
	No	478	56.9

Solid waste disposal	Open field	766	91.2
	In a pit	74	8.8

^*∗*^Multiple responses in %.

**Table 4:** Overall prevalence of malnutrition among under-five children in Dubti district, Afar Regional State, Northeast Ethiopia, 2017 (*n* = 840).

Anthropometric indices	Categories	Frequency (*n*)	Percent (%)
Weight for height (wasting)	Normal (≥−2WHZ score)	704	83.8
	Moderate wasting (−3 ≤ WHZ score < −2)	88	10.5
	Severe wasting (<−3WHZ score)	48	5.7
	Overall wasting (<−2WHZ score)	136	16.2

Height for age (stunting)	Normal (≥−2HAZ score)	478	56.9
	Moderate stunting (−3 ≤ HAZ score < −2)	222	26.4
	Severe stunting (<−3HAZ score)	140	16.7
	Overall stunting (<−2HAZ score)	362	43.1

Weight for age (underweight)	Normal (≥−2WAZ score)	632	75.2
	Moderate underweight (−3 ≤ WAZ score < −2)	98	11.7
	Severe underweight (<−3WAZ score)	110	13.1
	Overall underweight (<−2WAZ score)	208	24.8

**Table 5:** Factors associated with wasting among under-five children in Dubti district, Afar Regional State, Northeast Ethiopia, 2017 (*n* = 840).

Variables	Categories	Wasting		COR (95% CI)	AOR (95% CI)
		Yes (%)	No (%)		
Mother's occupation	Housewife	103 (15.2)	575 (84.8)	1	1
	Working	33 (20.4)	129 (79.6)	1.43 (1.12–2.65)	1.34 (0.84–2.14)

Family size	<5	25 (10.0)	224 (90.0)	1	1
	≥5	111 (18.8)	480 (81.2)	2.07 (1.31–3.29)	2.72 (1.62–4.55)^*∗*^

Place of delivery	Home	90 (18.6)	393 (81.4)	1.55 (1.05–2.28)	1.17 (0.76–2.79)
	Health facility	46 (12.9)	311 (87.1)	1	1

Prelacteal feeding	Yes	77 (21.9)	275 (78.1)	2.04 (1.41–2.95)	3.81 (1.79–5.42)^*∗*^
	No	59 (12.1)	429 (87.9)	1	1

Initiation of breastfeeding	Within 1 hour	49 (13.9)	303 (86.1)	1	1
	Hours later	87 (17.8)	401 (82.2)	1.75 (0.51–1.09)	0.98 (0.61–3.58)

Diarrhoea in the last two weeks	Yes	30 (27.8)	78 (72.2)	2.27 (1.42–3.63)	4.57 (2.56–8.16)^*∗*^
	No	106 (14.5)	626 (85.5)	1	1

Presence of latrine	Yes	51 (14.1)	311 (85.9)	1	1
	No	85 (17.8)	393 (82.2)	1.32 (0.90–1.92)	1.65 (0.98–3.65)

^*∗*^Significant at *p* < 0.05; COR = crude odd ratio; AOR = adjusted odd ratio; CI = confidence interval.

**Table 6:** Factors associated with stunting among under five children in Dubti district, Afar Regional State, Northeast Ethiopia, 2017 (*n* = 840).

Variables	Category	Stunting		COR (95% CI)	AOR (95% CI)
		Yes (%)	No (%)		
Sex of child	Male	234 (49.2)	242 (50.8)	1.78 (1.35–2.36)	1.98 (1.46–2.72)^*∗*^
	Female	128 (35.2)	236 (64.8)	1	1

Age of child	6–11	60 (23.6)	194 (76.4)	1	1
	12–23	112 (48.3)	120 (51.7)	3.02 (2.05–4.45)	3.44 (2.24–5.29)^*∗*^
	24–35	82 (53.2)	72 (46.8)	3.68 (2.39–5.66)	3.58 (2.25–5.69)^*∗*^
	36–59	108 (54.0)	92 (46.0)	3.79 (2.54–5.67)	4.42 (2.79–6.94)^*∗*^

Timely complementary feeding started	Yes	34 (32.4)	71 (67.6)	1	1
	No	328 (44.6)	407 (55.4)	1.68 (1.09–2.59)	1.87 (0.97–3.53)

Child immunization	Yes	54 (21.4)	198 (78.6)	1	1
	No	308 (52.4)	280 (47.6)	4.03 (2.87–5.68)	3.34 (2.31–4.81)^*∗*^

Diarrhoeal in the last two weeks	Yes	55 (50.8)	53 (49.1)	1.44 (0.96–2.15)	1.45 (0.91–3.31)
	No	307 (41.9)	440 (58.1)	1	1

Fever in the last two weeks	Yes	109 (49.8)	110 (50.2)	1.44 (1.06–1.96)	1.37 (0.94–2.98)
	No	253 (40.7)	368 (59.3)	1	1

Presence of latrine	Yes	141 (39.0)	221 (61.0)	1	1
	No	221 (46.2)	257 (53.8)	1.35 (1.02–2.18)	1.16 (0.84–2.59)

^*∗*^Significant at *p* < 0.05; COR = crude odd ratio; AOR = adjusted odd ratio; CI = confidence interval.

**Table 7:** Factors associated with underweight among under-five children in Dubti district, Afar Regional State, Northeast Ethiopia, 2017 (*n* = 840).

Variables	Category	Underweight		COR (95% CI)	AOR (95% CI)
		Yes (%)	No (%)		
Mother's occupation	Housewife	156 (23.0)	522 (77.0)	1	1
	Working	52 (32.1)	110 (67.9)	1.48 (1.08–2.30)	1.68 (0.87–3.38)

Mother education	Illiterate	197 (27.1)	529 (72.9)	3.49 (1.83–6.63)	4.06 (2.01–8.19)^*∗*^
	Educated	11 (9.6)	103 (90.4)	1	1

Child's sex	Male	149 (31.3)	327 (68.7)	2.36 (1.67–3.31)	1.83 (1.29–2.94)^*∗*^
	Female	59 (16.2)	305 (83.8)	1	1

Initiation of breastfeeding	Within one hour	77 (21.9)	275 (78.1)	1	1
	Hours later	131 (26.8)	357 (73.2)	1.31 (0.95–1.81)	0.82 (0.57–2.23)

Child immunization	Yes	29 (11.5)	223 (88.5)	1	1
	No	179 (30.4)	409 (69.6)	3.37 (2.00–5.15)	3.17 (2.14–7.99)^*∗*^

Ownership of livestock	Yes	171 (23.6)	553 (76.4)	1	1
	No	37 (31.9)	79 (68.1)	1.52 (0.99–2.32)	1.78 (0.92–4.54)

Prelacteal feeding	Yes	126 (35.8)	226 (64.2)	2.76 (1.04–3.81)	2.81 (1.64–3.72)^*∗*^
	No	82 (16.8)	406 (83.2)	1	1

^*∗*^Significant at *p* < 0.05; COR = crude odd ratio; AOR = adjusted odd ratio; CI = confidence interval.

## Data Availability

The data used to support the findings of this study are available from the corresponding author upon request.

## Abbreviations

EBF: Exclusive breastfeeding
EDHS: Ethiopian Demographic and Health Survey
FANTA: Food and Nutrition Technical Assistance
HAZ: Height-for-age *Z* score
HFIAS: Household Food Insecurity Access Scale
IQ: Intellectual quotient
IYCF: Infant and young child feeding
RERC: Research and Ethical Review Committee
SD: Standard deviation
WAZ: Weight-for-age *Z* score
WHO: World Health Organization
WHZ: Weight-for-height *Z* score.
